# Heterogeneous associations of cumulative heat exposure with cognitive decline among older Japanese adults

**DOI:** 10.1002/alz.71670

**Published:** 2026-07-14

**Authors:** Hiroyuki Hikichi, Kai Chen, Nobutoshi Nawa, Ayako Morita, Yusuke Matsuyama

**Affiliations:** ^1^ Department of Public Health Kitasato University School of Medicine Sagamihara Kanagawa Japan; ^2^ Department of Environmental Health Sciences Yale School of Public Health New Haven Connecticut USA; ^3^ Yale Center on Climate Change and Health Yale School of Public Health New Haven Connecticut USA; ^4^ Department of Public Health, Graduate School of Medical and Dental Sciences Institute of Science Tokyo Tokyo Japan; ^5^ Center for Well‐being Research Advancement Institute of Science Tokyo Tokyo Japan; ^6^ Department of Dental Public Health, Graduate School of Medical and Dental Sciences Institute of Science Tokyo Tokyo Japan

**Keywords:** cognitive decline, extreme heat, heterogeneity, older adults, wet bulb globe temperature

## Abstract

**INTRODUCTION:**

We investigated heterogeneity in the association between cumulative heat exposure and cognitive decline among older Japanese adults.

**METHODS:**

We analyzed 33,877 adults aged ≥ 65 years from a nationwide cohort (2010–2023) linked with long‐term care insurance data. Cumulative heat exposure was indexed using population‐weighted wet‐bulb globe temperature. Generalized random forests estimated the average and conditional treatment effects. Heterogeneity was decomposed using permutation importance, excess CATE analysis, and UpSet plot analyses.

**RESULTS:**

During a 12‐year follow‐up, 25.3% developed cognitive decline. Heat exposure increased incidence by 12.17 cases per 1000 person‐years (95% confidence interval [CI]: 2.54–21.62). Vulnerability profiles differed by age: among adults aged 65–74 years, multidimensional social and health constraints predominated; among those aged ≥ 75 years, functional decline and relational depletion dominated.

**DISCUSSION:**

Age‐specific vulnerability profiles provide a foundation for precision public health: multidomain interventions for adults aged 65–74 years and proactive social outreach for those aged ≥ 75 years.

## Highlights

- Cumulative heat exposure increased cognitive decline risk.
- Substantial individual heterogeneity was detected.
- Vulnerability patterns differed markedly by age group.
- Adults aged 65–74 years showed multidimensional constraint profiles.
- Adults aged ≥ 75 years showed functional and relational depletion.

## 1 BACKGROUND

With global warming intensifying, extreme heat events have become longer, more frequent, and more severe. Older adults are particularly vulnerable to heat exposure because of impaired thermoregulation and the high prevalence of chronic diseases^1^. Heat‐related mortality among older individuals has increased 2.67‐fold since the 1990s.^2^

Recent studies have suggested that heat exposure is associated not only with increased risks of acute illnesses, such as heat stroke and cardiac death,^3^ but also with heightened risks of chronic diseases, particularly cognitive decline among older adults.^4^

Epidemiological evidence for this association comes from several longitudinal studies. For instance, a longitudinal study of 9448 community‐dwelling American adults aged 50 years and older reported that long‐term exposure to extreme heat was linked to cognitive impairment among non‐Hispanic Black individuals and residents of socioeconomically disadvantaged neighborhoods.^5^ Similarly, a longitudinal study of approximately 30,000 Black and White adults aged 45 years and older found that long‐term heat exposure was associated with cognitive decline among individuals with lower educational attainment.^6^ These studies suggest that heat exposure is adversely associated with cognitive function, but considerable individual differences exist in the magnitude of this association.

**RESEARCH IN CONTEXT**

1. **Systematic review**: The authors searched PubMed for studies on heat exposure and cognitive decline published through January 2026. Although prior research has suggested an association between heat exposure and cognitive impairment, few studies have examined individual‐level heterogeneity in susceptibility, and none have characterized configurations of multidimensional vulnerability using causal machine‐learning approaches.
2. **Interpretation**: Cumulative heat exposure was associated with increased risk of cognitive decline, with substantial heterogeneity across individuals. Vulnerability patterns differed by age: among adults aged 65–74 years, risk reflected multidomain accumulation of social isolation, unemployment, physical inactivity, and chronic disease; among those aged ≥ 75 years, susceptibility was more concentrated and driven by functional decline and relational depletion.
3. **Future directions**: As climate change intensifies heat exposure, the population experiencing heat‐related health issues will grow, and the diversity of vulnerability profiles will expand alongside it. Future research should elucidate the pathways through which compound vulnerability profiles confer susceptibility, and evaluate whether interventions targeting these profiles can reduce heat‐related cognitive decline—advancing the precision public health goal of directing resources toward those at highest risk.

As rising temperatures become more prolonged and widespread, their health consequences are shaped by where people live, their socioeconomic circumstances, and their underlying health status.^7^ Uniform public health warnings are unlikely to reach those at greatest risk, because population‐average approaches cannot identify which subgroups are most vulnerable.^8^ Dementia further compounds this challenge, as its etiology is multifactorial and its risk is shaped by the intersection of multiple domains rather than any single cause.^9^ A precision public health approach, which uses individual‐level data and advanced analytical methods to identify actionable subpopulations, offers a principled solution to this problem.

Conventional analyses are poorly suited to this task. Researchers typically formulate hypotheses about potential effect modifiers in advance and introduce interaction terms into regression models one at a time. This deductive approach cannot detect higher‐order interactions that emerge from combinations of multiple variables, which may lead to the omission of important effect modifiers.^10^ Testing multiple interaction terms also raises concerns about multiple comparisons and model overfitting.^10^ In contrast, causal machine learning methods, such as the Generalized Random Forest (GRF), overcome these limitations by estimating Conditional Average Treatment Effects (CATEs), individual‐level causal effect estimates, while simultaneously accounting for intercorrelations among multiple variables.^11^, ^12^ This data‐driven approach facilitates the identification of vulnerable subgroups whose risk of cognitive decline may be disproportionately elevated under heat stress.

To operationalize this approach, we applied the GRF algorithm to estimate the association between cumulative heat exposure and cognitive decline, and its heterogeneity, using data from an 11‐year cohort study of Japanese older adults. We then identified combinations of factors that amplify individual vulnerability to heat‐related cognitive decline.

## 2 METHODS

### 2.1 Study participants

Between August 2010 and December 2011, the Japan Gerontological Evaluation Study (JAGES) conducted baseline surveys assessing the health and lifestyle of community‐dwelling adults aged 65 years or older who were physically and cognitively independent. JAGES is a nationwide cohort study covering eight municipalities that represent both urban and rural settings. Two municipalities were subsequently excluded: one because cognitive function data were unavailable for more than 3 follow‐up years, and the other because the 2011 Great East Japan Earthquake and Tsunami caused radical environmental changes in the residential area.

Using municipal residential registers, JAGES carried out complete enumeration surveys in three municipalities and random‐sample surveys in the remaining three. Of 60,994 eligible residents, 38,393 individuals responded (response rate = 62.9%). Of these, 33,956 participants were successfully linked to the Long‐Term Care Insurance (LTCI) database, which was used to ascertain cognitive decline. We further excluded 79 participants with missing elementary school district codes, which were required to link meteorological data, yielding a final analytic sample of 33,877.

Across the six municipalities, follow‐up durations ranged from 3984 to 4572 days, with a median of 4220 days. The latest observation period ended on March 30, 2023.

After the first diagnosis of cognitive decline, the participants were excluded to avoid counting post‐outcome exposure time. We additionally censored observations that ended in death (*n* = 5954) or attrition due to relocation (*n* = 842) prior to cognitive decline onset.

The survey protocol was reviewed and approved by the Ethics Committees on Research of Human Subjects at Nihon Fukushi University (10–05). Each respondent provided informed consent to participate in the study.

### 2.2 Explanatory variable

The explanatory variable was the Wet Bulb Globe Temperature (WBGT), a composite index that integrates natural wet bulb temperature, globe temperature, and dry bulb temperature. WBGT accounts for humidity, ambient air temperature, radiant heat, and wind speed.^13^ WBGT was selected over air temperature alone because composite indices incorporating humidity have shown stronger associations with heat‐related morbidity and mortality than air temperature‐based definitions in a humid climate context.^14^

We obtained 1‐km grid square meteorological data from the National Agriculture and Food Research Organization and calculated WBGT using the following equation^15^:

$$\begin{array}{ccc} \mathrm{WBGT} & = & 0.735\times \mathrm{Ta}+0.0374\times \mathrm{RH}+0.00292\times \mathrm{Ta}\times \mathrm{RH}\\ & & +\;7.619\times \mathrm{SR}-4.557\times {\mathrm{SR}}^{2}-0.0572\times \mathrm{WS}-4.064 \end{array}$$

where Ta is dry‐bulb temperature (°C), RH represents relative humidity (%), SR represents global solar radiation (kW/m^2^), and WS is wind speed (m/s).

This equation has been validated against meteorological station data from six urban cities in Japan, demonstrating high estimation accuracy with errors below 1°C and confidence levels ranging from 98.3% to 99.8%.^15^

Daily 1‐km WBGT raster data were spatially linked to elementary school district polygons obtained from the National Land Numerical Information database using the sf in R package, because JAGES uses elementary school districts as the smallest geographic unit within each municipality. District‐level WBGT was calculated as the population‐weighted mean of all intersecting 1‐km grid cells. Population weights were derived from a 500‐m mesh of the 2010 census, and partial overlaps between grid cells and district polygons were allocated proportionally by area.

To construct the Extreme Heat Index (EHI), we first calculated the 80th, 85th, and 90th percentile thresholds of daily WBGT using data from May to September across all years from 2010 to 2023. For each day *d* and percentile *p*, we computed the daily exceedance as

$$\;\delta_{d,\;p}=\max\left(\mathrm{WBG}T_{d}-\tau_{p},\;0\right)$$

where $\tau_{p}$ denotes the *p*th‐percentile WBGT. We then aggregated these positive exceedances within each calendar month *m* to derive the monthly EHI:

$$\;EHI_{m,\;p}=\sum_{d\in m}\delta_{d,\;p}$$

This approach yields a continuous metric that captures both the frequency and intensity of heat days exceeding each percentile threshold.

### 2.3 Outcome variable

The outcome was cognitive decline, identified using the LTCI database. Since 2001, the Japanese government has operated a national insurance system providing long‐term care services to older adults in need. Trained investigators assess cognitive function, including short‐term memory, orientation, and communication abilities, as well as mental and behavioral symptoms, such as persecutory delusions and confabulation, using a standardized in‐home evaluation protocol.^16^ Based on these evaluations, applicants are classified into one of seven levels of cognitive disability, ranging from Level 1 (mild cognitive deficits with largely preserved independence) to Level 7 (requiring continuous medical care in a specialized facility) (Table S1).^17^ This index is strongly correlated with the Mini‐Mental State Examination (Spearman's *ρ* = 0.73, *p* < 0.01).^18^ Furthermore, Level 1 on this scale has been shown to correspond to a score of 0.5 on the Clinical Dementia Rating, with both sensitivity and specificity of 0.88.^19^

For the analysis, we defined cognitive decline as a binary outcome using a cutoff at Level IIa of the cognitive disability scale.^20^, ^21^ Level IIa indicates loss of independence in daily life, characterized by frequently getting lost in familiar areas or making noticeable errors in tasks previously managed independently, such as shopping, personal administration, or financial management.

### 2.4 Covariates

We selected 52 potential confounders at baseline across seven domains (Table S2), including four demographic characteristics (age, sex, marital status [divorced or bereaved], and living alone); four socioeconomic characteristics (low educational attainment, low equivalized household income, unemployment, and no home ownership); 21 health conditions (activities of daily living, depressive symptoms [Geriatric Depression Scale–15 score ≥ 5],^22^ and current treatment for 19 medical conditions including cancer, heart disease, stroke, hypertension, diabetes, hyperlipidemia, arthritis or neuralgia, injury or fracture, respiratory disease, visual impairment, hearing impairment, hepatic disease, gastrointestinal disease, obesity, osteoporosis, mental disorders, urination disorders, sleep disorders, and dysphagia); eight social relationship factors (lack of emotional and instrumental support [both giving and receiving], infrequent participation in hobby or sports clubs, infrequent meetings with friends, and limited daily interaction with neighbors); six behavioral factors (current smoking, current alcohol use, infrequent consumption of meat or fish, infrequent consumption of vegetables or fruits, infrequent outings, and short daily walking time); three adverse life events within the past year (loss of a spouse, loss of relatives or friends, and initiation of family caregiving); and six neighborhood environmental factors (presence of graffiti or litter, less parks and sidewalks suitable for exercise, difficult terrain due to hills or steps, roads or intersections with a high risk of traffic accidents, less attractive scenery or buildings, and unsafe conditions for walking alone at night) (see also Table S2).

### 2.5 Statistical analysis

The analytical approach proceeded in five stages. First, we used Distributed Lag Non‐Linear Models (DLNMs) to identify the optimal lag structure and heat exposure specification. Second, we estimated the ATE and CATE of cumulative heat exposure on cognitive decline using GRF with an R‐learner framework. In the remaining three stages, we explored components of heat‐related vulnerability within the higher stratum of CATE, defined as above the 80th percentile of the positive CATE distribution. Third, we applied permutation importance analysis to identify variables that discriminate individuals above the higher stratum of CATE from those with the lower estimates. Fourth, within this high‐vulnerability stratum, we estimated the excess CATE above the 80th percentile threshold for each identified attribute. Fifth, we used UpSet plots to characterize the co‐occurrence patterns of vulnerability attributes within the same stratum.

In a preliminary analysis, we identified the optimal lag structure and exposure specification using DLNMs fitted to person‐month data separately for each municipality and pooled via multivariate random‐effects meta‐analysis.^23^, ^24^ We compared candidate models across WBGT exceedance thresholds (80th, 85th, and 90th percentiles), maximum lag periods (12–36 months), and spline complexity (2–4 degrees of freedom). Model selection was based on the quasi‐Akaike information criterion.^23^ The final specification comprised the monthly sum of population‐weighted WBGT exceedances above the 80th percentile, a 24‐month lag window. These parameters were carried forward to construct the cumulative exposure metric in the primary analysis. Full details of DLNMs are provided in the Supplementary Methods.

In the primary analysis, we employed GRF with an R‐learner framework to estimate the ATE and CATE of cumulative heat exposure on cognitive decline using person‐month data. GRF extends random forest algorithms to estimate heterogeneous treatment effects across subpopulations defined by observed covariates.^12^ The causal estimand was defined as the expected change in the probability of cognitive decline associated with a one interquartile range (IQR) increase in cumulative heat exposure, corresponding to a shift from the 25th to the 75th percentile of its distribution in the study population. We constructed a cumulative heat exposure metric as the monthly sum of population‐weighted WBGT exceedances above the 80th percentile over the preceding 24 months (t−1 to t−24). To emphasize recent exposure while incorporating cumulative effects, we summarized this metric using an exponentially weighted moving average (EWMA) with a half‐life of 12 months, assigning weights $\;W_{k}\propto \;2^{-k/12}$ normalized to sum to 1. This parameterization reflects evidence that heat‐related health effects are concentrated in more proximal exposure periods.^25^ To accommodate continuous heat exposure, we used regression forests with cross‐fitted nuisance functions.^26^ We implemented honest splitting, in which separate subsamples were used for tree construction and effect estimation to reduce overfitting.^11^ To account for intra‐individual correlation during model training, cross‐fitting folds were assigned at the individual level, ensuring that all person‐month observations from the same individual were allocated to the same fold. These analyses were conducted using the grf package.

To account for competing risks from death and loss to follow‐up prior to cognitive decline, we estimated inverse probability of censoring weights (IPCW) from baseline covariates, including age, sex, educational attainment, equivalized household income, employment status, marital status, depressive symptoms, and comorbidities (heart disease and stroke). Stabilized weights were then incorporated into the forest model.

We included seasonal terms and a long‐term time trend in both the IPCW models and nuisance function models to separate temporal variation from heat exposure effects. Municipality indicators were included as covariates to account for geographic differences. Statistical inference was conducted using cluster bootstrap resampling with individuals as the sampling unit (*B* = 10,000) to account for within‐person correlation across repeated monthly observations.^27^

All post‐hoc analyses were conducted at the person level, using each individual's IPCW‐weighted means of the estimated treatment effect, outcome residual, and exposure residual aggregated across person‐month observations, to avoid inflation of test statistics from repeated measures.

Although age was modeled as a continuous covariate in the GRF estimation stage to maximize precision, heterogeneity decomposition was conducted separately for two policy‐relevant age strata: 65–74 years (*n* = 20,107) and ≥ 75 years (*n* = 13,770). This stratification reflects a meaningful institutional boundary in Japan, where adults aged 75 years and older transition to a separate insurance scheme with distinct cost‐sharing requirements.^28^

To characterize vulnerability profiles across the CATE distribution, we implemented a permutation importance (PI) analysis using the DALEX package. The analysis was restricted to individuals with a positive estimated treatment effect (CATE > 0), thereby focusing on heterogeneity in susceptibility rather than in effect direction. Within each age stratum, individuals were classified as high‐vulnerability (*y* = 1) if their person‐level mean CATE exceeded the stratum‐specific 80th percentile, and as lower‐vulnerability (*y* = 0) otherwise. Unlike traditional importance measures based on impurity reduction or mutual information, which favor variables with many categories or high variability,^29^ PI quantifies feature relevance by randomly permuting each variable's values and measuring the resulting decrease in model discrimination.^30^ We used the reduction in the area under the receiver operating characteristic curve (ΔAUC) as the importance metric, where larger reductions indicate stronger contributions to distinguishing high‐ from lower‐vulnerability individuals.^31^

After the PI analysis, we selected the top 15 attributes identified within each age stratum for detailed characterization of the high‐vulnerability subpopulation (CATE ≥ 80th percentile). To enable interpretable comparisons, we binarized all covariates prior to subsequent analyses. Binary variables, such as sex and disease diagnoses, remained unchanged. Ordinal and continuous variables were dichotomized at the 67th percentile, designating the upper tertile as high‐risk and the lower two tertiles as low‐risk.

For each selected attribute, we estimated the difference (Δ) between the mean CATE among individuals with that attribute and the stratum‐specific 80th percentile CATE threshold. A positive Δ indicates that individuals with the attribute had higher heat susceptibility than the threshold defining the high‐vulnerability stratum. Uncertainty was assessed using person‐level bootstrap resampling (500 replications), with the 80th percentile threshold held fixed across replications.

To describe co‐occurring vulnerability profiles within the high‐vulnerability subpopulation, we used UpSet plots implemented via the ComplexUpset package to visualize the frequency and mean excess CATE of multi‐attribute combinations among the top 15 PI‐selected attributes. The analysis was restricted to combinations involving two or more attributes and occurring in at least 30 individuals. For each combination, mean ΔCATE above the 80th percentile threshold was calculated and displayed alongside intersection size, enabling simultaneous assessment of combination prevalence and heat susceptibility magnitude. This approach enabled identification of the most prevalent composite vulnerability profiles rather than isolated risk factors.^32^

In a sensitivity analysis, we extended the lag window to 36 months, informed by prior evidence suggesting that cumulative heat exposure over a three‐year period may be associated with cognitive decline among Japanese older adults,^33^ to examine whether findings were robust to longer exposure windows.

Missing data were imputed using a random forest algorithm implemented using the missForest package.^34^ To assess the robustness of the ATE estimate and CATE distribution to the choice of missing data handling strategy, a sensitivity analysis was conducted using multiple imputation with chained equations using random forests (MICErf; *m* = 10),^35^ with results pooled via Rubin's rules. All analyses were conducted using R version 4.5.0 (R Foundation for Statistical Computing, Vienna, Austria).

## 3 RESULTS

Table 1 presents descriptive statistics for the study sample (*n* = 33,877). During the follow‐up period, 8571 individuals (25.3%) developed cognitive decline. The mean age was 73.8 years (SD = 6.01), and 53.4% were female. At baseline, 25.2% were divorced or widowed, and 13.9% were living alone.

**TABLE 1:** Characteristics of our analytic sample (*n* = 33,877).

Parameter	*n* / %	Mean / SD
Outcome		
Incidence of cognitive decline (≧ IIa), *n* (%)	8571 (25.3)	
Baseline demographic characteristics		
Age, years, mean (SD)		73.80 (6.01)
Female, *n* (%)	18,080 (53.4)	
Divorce/bereavement, *n* (%)	8542 (25.2)	
Missing, *n* (%)	550 (1.6)	
Living alone, *n* (%)	4725 (13.9)	
Missing, *n* (%)	559 (1.7)	
Baseline socioeconomic characteristics		
Low educational attainment (1: ≧ 13y to 4: < 6y), mean (SD)		2.25 (0.79)
Missing, *n* (%)	899 (2.7)	
Low equivalized household income, mean (SD)^a^		6.83 (2.43)
Missing, *n* (%)	6191 (18.3)	
Unemployment, *n* (%)	23,021 (70.0)	
Missing, *n* (%)	3965 (11.7)	
No home ownership, *n* (%)	4711 (13.9)	
Missing, *n* (%)	633 (1.9)	

^a^ We divided the household's gross income by the square root of the number of household members, and created nine categories using 0.5‐million‐yen intervals (≤1.5 million yen, 1.5–2.0 million yen, …, ≥5.0 million yen). In this study, the nine‐category income scale was reverse‐coded so that higher values indicated lower income.

We also summarized baseline health conditions in Table S3. At baseline, 22.5% of participants exhibited a higher risk of depressive symptoms, and 38.7% of them had hypertension under medical treatment.

Figure S1 presents the distribution of school district–level median monthly EWMA of WBGT exceedances above the district‐specific 80th percentile. Between‐municipality variability exceeded within‐municipality variability in this exposure metric, justifying adjustment for geographic differences in the conditional treatment estimation.

The GRF analysis showed an ATE of 12.17 cases per 1000 person‐years for a one‐IQR increase in 24‐month EWMA‐weighted cumulative heat exposure (95% CI: 2.54, 21.62) (Table 2). The IQR was calculated within each municipality and averaged equally across municipalities to prevent the overall estimate from being disproportionately driven by areas with larger sample sizes. The CATE estimates were centered near zero (mean: −0.0008, median: 0.0004, SD: 0.130); the interquartile range spanned −0.0106 to 0.0110. Post‐hoc calibration testing^36^ confirmed that individuals predicted to be more susceptible to heat exposure had higher observed rates of cognitive decline (*β* = 1.589, SE = 0.034; one‐sided *p* < 0.001). This result indicates that the estimated heterogeneity reflects genuine differences in susceptibility across individuals. The Group Average Treatment Effects (GATES)^36^ analysis showed a monotonic gradient in group‐specific effects (Figure S2): the lowest CATE group (G1) showed negative effects (*β* = −0.142) and the highest group (G5) showed positive effects (*β* = 0.119). The GATES spread test rejected equality of group effects (*χ* ^2^ = 4,506.3, df = 4, *p* < 0.001), supporting robust heterogeneity across the CATE distribution. A sensitivity analysis using MICErf (m = 10) yielded consistent results in both the ATE estimate (9.59 cases per 1,000 person‐years, 95% CI: 0.29, 18.89) and the GATES monotonic gradient (G1: *β* = −0.114; G5: *β* = +0.100; spread test: *χ* ^2^ = 4,577.5, df = 4, *p* < 0.001).

**TABLE 2:** Association between cumulative heat exposure and cognitive decline.

Parameter	Cases per 1000 person‐years (95% CI)
Per municipality‐specific IQR increase in cumulative heat exposure over the preceding 24 months (EWMA‐weighted)^a^	12.17 (2.54, 21.62)

^a^ IQR was calculated within each municipality. The effect estimates were averaged equally across municipalities to prevent the overall estimate from being disproportionately driven by areas with larger sample sizes.

Abbreviations: CI, confidence interval; EWMA, exponentially weighted moving average; IQR, interquartile range.

**FIGURE 1:**
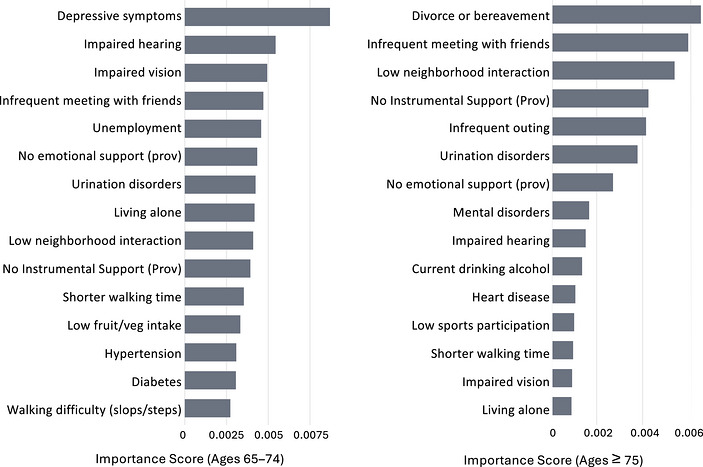
Permutation importance of vulnerability factors for high heat susceptibility (CATE ≥ 80th percentile), by age stratum. Note: Importance scores represent the decrease in area under the receiver operating characteristic curve (ΔAUC) when each variable is randomly permuted. Higher values indicate greater discriminative contribution to identifying individuals above the 80th percentile of the positive‐CATE distribution. Abbreviation: CATE, Conditional Average Treatment Effect; ΔAUC, change in area under the receiver operating characteristic curve.

Permutation importance analyses revealed distinct vulnerability profiles across age strata (Figure 1). Among participants aged 65–74 years (AUC = 0.68), depressive symptoms showed the strongest contribution to identifying high‐vulnerability individuals, followed by sensory impairments (hearing and vision), infrequent social contact, unemployment, and absence of instrumental support. This pattern suggests that high heat susceptibility in younger‐old adults is distributed across multiple domains, including mental health, functional capacity, and social integration. Among participants aged 75 years and older (AUC = 0.62), the leading discriminating factors were marital loss or bereavement and reduced outdoor activity, alongside infrequent social contact, low neighborhood interaction, and urination disorders. In this group, social disconnection and geriatric conditions dominated, whereas the broader spread of domains observed in the younger age stratum was less evident.

**FIGURE 2:**
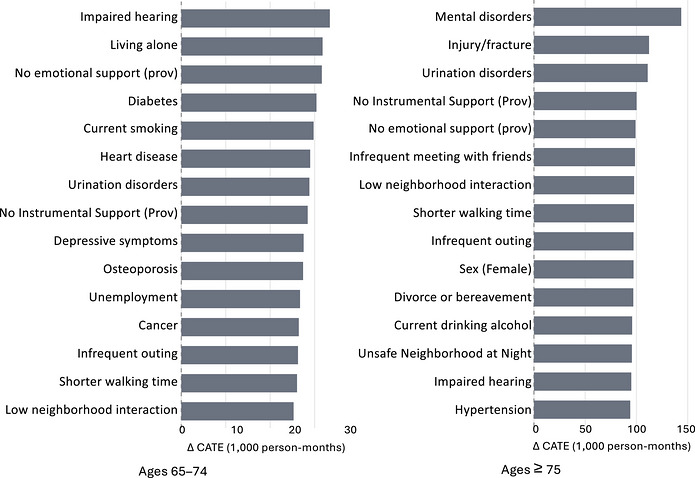
Excess CATE above the 80th percentile threshold for the top 15 vulnerability attributes, by age stratum. Note: The mean excess CATEs (Δ) estimated within the high‐vulnerability subpopulation (CATE ≥ 80th percentile). CATE, Conditional Average Treatment Effect.

Within the high‐CATE stratum (≥ 80th percentile), all 15 selected attributes showed positive Δ values in both age groups (Figure 2). Among participants aged 65–74 years, excess CATE above the threshold was distributed narrowly across attributes (range: 25–33 per 1000 person‐months), spanning sensory, social, metabolic, and functional domains. Among participants aged 75 years and older, most attributes clustered within a narrow range (range: 94–100 per 1000 person‐months). Mental disorders and injury or fracture showed the largest values (Δ = 144 and 112 per 1000 person‐months, respectively).

**FIGURE 3:**
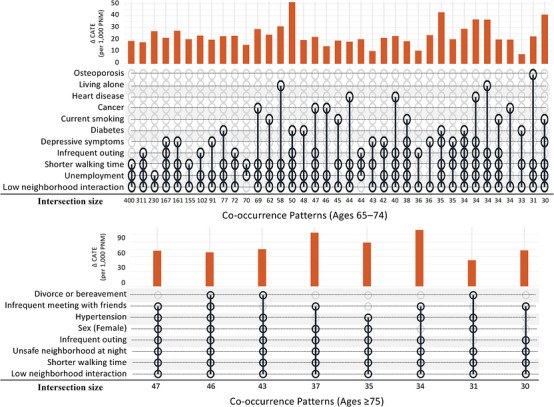
Co‐occurrence patterns of vulnerability attributes and mean excess CATE within the high‐vulnerability subpopulation (CATE ≥ 80th percentile), by age stratum. CATE, Conditional Average Treatment Effect; PNM, person‐months.

UpSet plots revealed distinct co‐occurrence patterns between the two age groups (Figure 3). Among participants aged 65–74 years, 37 combinations met the minimum support threshold (*n* ≥ 30), with intersection sizes ranging from 30 to 400. The most frequent combination comprised low neighborhood interaction, unemployment, and shorter walking time (*n* = 400). Low neighborhood interaction appeared in nearly all identified combinations, functioning as a common anchor across diverse co‐occurring patterns. Additional factors incorporated across combinations included depressive symptoms, diabetes, cancer, heart disease, and infrequent outings, spanning metabolic, mental health, and functional domains. The highest mean excess CATE was observed for a five‐factor combination of low neighborhood interaction, unemployment, shorter walking time, infrequent outings, and diabetes (Δ = 51.4 per 1000 person‐months).

Among participants aged 75 years and older, eight combinations were identified (intersection sizes: 30–47), all sharing a common core of low neighborhood interaction, shorter walking time, and an unsafe neighborhood environment at night. The highest mean excess CATE was observed for a six‐factor combination of the core plus infrequent outing, infrequent meeting with friends, and hypertension (Δ = 114.2 per 1000 person‐months).

As an additional analysis, we investigated the origin of negative CATEs (CATE < 0) to determine whether they reflect genuine protective effects or arise from data structure. R‐learner components were compared across three CATE strata (negative, near‐zero, and positive). The predicted outcome (m ^) and outcome residual (Y ~) showed no meaningful differences across strata. The exposure residual (T ~) differed markedly, with a median of +0.018 in the negative CATE group and −0.018 in the positive CATE group (Table S4). Within the negative CATE group, participants were further divided by the sign of T ~. The below‐zero T ~ subgroup (*n* = 3161) reached a cumulative incidence of cognitive decline exceeding 0.80 by end of follow‐up, whereas the above‐zero T ~ subgroup (*n* = 9621) remained below 0.20 (Figure S3). The below‐zero T ~ subgroup was older and showed higher prevalence of infrequent outing, urination disorders, divorce or bereavement, lack of instrumental social support, shorter walking time, unemployment, and no homeownership (all standardized mean differences [SMDs] > 0.10;^37^ Figure S4).

Sensitivity analyses using a 36‐month lag specification yielded consistent findings. The ATE was 16.29 cases per 1000 person‐years (95% CI: 4.61, 27.59). Effect heterogeneity was confirmed by calibration testing (*β* = 1.654, SE = 0.031, *p* < 0.001) and GATES analysis (*χ* ^2^ = 4920.2, df = 4, *p* < 0.001) (Figure S5). The CATE was centered near zero (mean: −0.0002, median: 0.0004, SD: 0.152). Fixed‐window permutation importance analyses, excess CATE analysis, and UpSet plots showed similar patterns across both lag specifications, supporting the robustness of the identified vulnerability profiles (Figures S6–S8).

## 4 DISCUSSION

This study demonstrated the association between cumulative heat exposure and cognitive decline onset, with a primary focus on heterogeneity in susceptibility, using a large‐scale population‐based cohort linked to long‐term care insurance records. The GRF yielded a significant ATE of 12.17 cases per 1000 person‐years for heat exposure over the preceding 24 months (EWMA‐weighted), which increased to 16.29 cases per 1000 person‐years in the 36‐month lag sensitivity analysis.

To examine heterogeneity in heat susceptibility, we conducted a four‐step analysis in each age stratum. First, GRF demonstrated significant individual variation in CATEs, with a monotonic gradient across CATE quantile groups confirmed by GATES analysis. Second, permutation importance analyses identified age‐specific vulnerability profiles: among adults aged 65–74 years, contributing attributes spanned mental health, sensory, social, and functional domains, whereas among adults aged 75 years and older, social disconnection and geriatric conditions predominated. Third, within the high‐vulnerability stratum (CATE > 0, ≥ 80th percentile), excess CATE above the threshold was distributed narrowly across all 15 attributes among adults aged 65–74 years, with no single factor showing clear dominance. Among adults aged 75 years and older, mental disorder and fracture showed notably higher excess CATE values relative to other attributes, suggesting that severe geriatric conditions contribute disproportionately to high susceptibility in this age group. Fourth, co‐occurrence analysis revealed that vulnerability profiles were structured as compound configurations rather than isolated risk factors. Among adults aged 65–74 years, low neighborhood interaction served as a common anchor across 37 recurrent combinations, with additional factors spanning metabolic, mental health, and functional domains. Among adults aged 75 years and older, eight combinations were identified, all sharing a concentrated core of social and functional constraints. These findings suggest that heat susceptibility reflects the combined influence of multiple factors, and that the composition of these profiles differs markedly by age stratum.

Among younger‐old adults, no single factor dominated vulnerability. Instead, reduced social interaction, physical inactivity, and unemployment formed a cross‐domain core, and the addition of chronic conditions such as diabetes or depressive symptoms further amplified heat susceptibility. This pattern suggests that vulnerability at this life stage reflects the simultaneous accumulation of constraints across multiple domains rather than any single predominant pathway. Among older‐old adults, single factors such as mental disorders, injury or fracture, and urination disorders independently elevated heat susceptibility to a substantial degree. Co‐occurrence patterns were less diverse than in younger‐old adults, yet a concentrated core of social and physical constraints including infrequent outings, reduced mobility, and unsafe neighborhood environments remained consistently present. In the Japanese context, unsafe neighborhood environments likely reflect physical barriers such as insufficient street lighting, uneven pavements, and pedestrian bridges rather than neighborhood crime, given the functional limitations characteristic of this age group.

These age‐specific profiles imply differentiated intervention priorities. For younger‐old adults, multidomain strategies are indicated, including policies that support continued workforce participation, structured social engagement, and promotion of physical activity. For older‐old adults, both active and proactive approaches are warranted. Those retaining social capacity may benefit from community participation programs, whereas individuals with reduced mobility or social isolation require proactive outreach such as regular home visits, telephone‐based wellness checks, or municipal monitoring services to identify high‐risk individuals before adverse events occur.

The prominence of social disconnection as a discriminator across both age strata aligns with findings from the 1995 Chicago heat wave, in which socially isolated individuals experienced disproportionately high mortality.^38^ Our study extended this evidence to cognitive outcomes and further identified the specific combinations of attributes that amplify heat‐related vulnerability beyond social disconnection alone.

The observed negative CATEs warrant careful interpretation. An analysis of R‐learner components revealed that neither the predicted outcome ($\hat{m}$) nor the outcome residual ($\tilde{Y}$) differed meaningfully across CATE strata, whereas the exposure residual ($\tilde{T}$) differed markedly (median: 0.018 in the negative CATE group and −0.018 in the positive CATE group). These results suggest that individuals in the negative CATE group received higher cumulative heat exposure than their covariate profile would predict. However, the negative CATE group was internally heterogeneous with respect to $\tilde{T}$, suggesting the presence of two mechanistically distinct subpopulations.

The cumulative incidence functions and baseline characteristics of the two $\tilde{T}$ subgroups support this interpretation. The below‐zero $\tilde{T}$ subgroup reached a cumulative incidence exceeding 0.80 by the end of follow‐up. This subgroup was older and showed a higher prevalence of social isolation, functional decline, and adverse life events, indicating early cognitive decline driven by accumulated vulnerability independent of heat. The above‐zero $\tilde{T}$ subgroup remained below a cumulative incidence of 0.20 despite above‐predicted heat exposure, reflecting maintained cognitive function under sustained thermal stress. Taken together, these findings suggest that negative CATEs reflect a mixture of survivorship bias and a healthy survivor effect rather than a genuine protective effect of heat exposure.

Our study has several notable strengths. First, we operationalized a precision public health approach using causal machine learning. By estimating CATEs at the individual level, we moved beyond population‐average effects to identify who is most susceptible to cumulative heat exposure. This individual‐level approach provides the empirical foundation for targeting interventions toward those most likely to benefit, rather than applying uniform strategies to entire populations. Second, studies applying causal machine learning to treatment effect heterogeneity have often focused on single effect modifiers. Although UpSet plots have increasingly been used in epidemiology to visualize co‐occurrence patterns alongside effect estimates,^39^ combining this approach with individual‐level CATE estimation to quantify treatment effect heterogeneity associated with each attribute combination appears to be uncommon. This specificity is what precision public health requires: intervention targets defined by the intersection of multiple characteristics rather than by any single factor.^8^ Third, the vulnerability profiles identified in this study translate directly into intervention priorities implementable within existing institutional frameworks, including community‐based dementia prevention initiatives,^40^ employment support for older workers,^41^ and integrated community care programs.^42^ Critically, our findings suggest that coordinating existing services across multiple domains simultaneously, rather than delivering them sequentially, is essential to reach individuals whose specific combination of attributes places them at the highest risk

This study also has several limitations. First, unmeasured factors, including air conditioner use, adequate hydration, and cognitive reserve, may have influenced the CATE estimates. Despite a systematic literature search, no peer‐reviewed method was identified for formally assessing residual confounding in CATEs estimated from continuous exposures using nonparametric learners. With respect to the negative CATE specifically, these unmeasured factors may have contributed to both mechanisms described above. Second, both the GRF covariates and the IPCW models relied on baseline measurements, and time‐varying confounding during the follow‐up period was not formally addressed. At baseline, however, the breadth of covariates spanning social, behavioral, environmental, and existing disease domains reduces the likelihood that major confounders were omitted. Third, cognitive decline was ascertained through LTCI certification records, which depend on individual application rather than systematic clinical screening. Since LTCI certification requires self‐initiation, the timing of certification may not accurately reflect the true onset of cognitive decline. The consistency of findings across both the primary analysis (24‐month lag) and the sensitivity analysis (36‐month lag) suggests that the observed associations are robust to variation in the timing of outcome ascertainment. Fourth, inter‐municipality variation in certification processes may have introduced heterogeneity in the outcome detection. This was partially addressed by including municipality indicators as covariates in the GRF model.

As global temperatures continue to rise, the population vulnerable to heat‐related health issues is expected to grow, and the diversity of vulnerability profiles is likely to expand alongside this. Differences in underlying disease burden, medication use, socioeconomic circumstances, and social support will produce increasingly heterogeneous patterns of susceptibility that population‐average approaches cannot capture. This is precisely the context in which a precision public health approach becomes essential. This study shows that susceptibility to cumulative heat exposure and cognitive decline among older adults follows age‐specific configurations of multidimensional vulnerability. Building on evidence that approximately 40% of dementia cases are attributable to modifiable risk factors,^9^ the impact of cumulative heat stress under a changing climate may represent an emerging modifiable determinant. The compound vulnerability profiles identified here may guide existing community health and social care systems in prioritizing multidomain interventions for those at highest risk.

## AUTHOR CONTRIBUTIONS

H.H. conceptualized the study, designed the methodology, and performed the statistical analyses. H.H. curated the data, interpreted the results, and drafted the manuscript. C.K., N.N., A.M., and Y.M. contributed to critical manuscript revision. All authors approved the final manuscript.

## CONFLICT OF INTEREST STATEMENT

Authors declare no competing interests. Author disclosures are available in the Supporting Information.

## CONSENT STATEMENT

All human subjects provided informed consent.

## Supporting information



Supporting information

Supporting information

## Data Availability

Restrictions apply to the availability of these data, which are used under agreements with the participating municipality. Data are available from the JAGES Secretariat upon reasonable request, pending committee approval and a data‐use agreement.
