# Supplementary material for: Heterogeneous associations of cumulative heat exposure with cognitive decline among older Japanese adults
Source: Alzheimers Dement. 2026 Jul 14;22(7):e71670. doi: 10.1002/alz.71670 (PMC13368698; doi:10.1002/alz.71670)
Supplement: Supplementary file 1 — Supporting information [file ALZ-22-e71670-s001.pdf]

Supplementary Information

Heterogeneous associations of cumulative heat exposure with cognitive decline among older Japanese adults

Authors: Hiroyuki Hikichi, PhD; Chen Kai, PhD; Nobutoshi Nawa, MD, PhD; Ayako Morita, PhD; and Yusuke Matsuyama, DDS, PhD

Corresponding author: Hiroyuki Hikichi, PhD

Email: hikichi@med.kitasato-u.ac.jp

Supplementary Tables and Figures

Table S1. Levels of cognitive decline in the Japanese long-term care insurance scheme

Table S2. Definitions of covariates

Table S3. Descriptive statistics of baseline covariates (not included in Table 1)

Figure S1. Spatial variability in school-district heat exposure within municipalities (EWMA 24-month; half-life = 12 months)

Figure S2. Group average treatment effects (GATES) across five CATE quantile groups

Table S4. R-learner component comparison across CATE strata

Figure S3. Cumulative incidence of cognitive decline by exposure residual subgroup within the negative CATE group

Figure S4. Baseline covariate balance between exposure residual subgroups within the negative CATE group

Figure S5. Group average treatment effects (GATES) across five CATE quantile groups: sensitivity analysis with a 36-month exposure lag

Figure S6. Permutation importance of vulnerability factors for high heat susceptibility (upper 20th percentile of CATE), by age stratum: sensitivity analysis with a 36-month exposure lag

Figure S7. Excess CATE above the 80th percentile threshold for the top 15 vulnerability attributes, by age stratum: sensitivity analysis with a 36-month exposure lag

Figure S8. Co-occurrence patterns of vulnerability attributes and mean excess CATE within the high-vulnerability subpopulation (CATE ≥ 80th percentile), by age stratum: sensitivity analysis with a 36-month exposure lag

Supplementary Methods

・Full details of Distributed Lag Non-Linear Models

Table S1. Levels of cognitive decline in the Japanese long-term care insurance scheme

Rank		Criteria	Examples of observable symptoms or behaviors
I		Suffers from a certain cognitive decline, but the daily living is almost all independent in the domestic and social spheres.	
II		Manifests some symptoms/behaviors and communication difficulties that may hinder the daily activities, but can be independent if someone takes care them.	
	IIa	The abovementioned conditions in II are observed while outside the domestic sphere.	Frequently gets lost on the street, or makes noticeable mistakes in matters that the person was previously able to handle, such as shopping, personal administrative tasks, or financial management.
	IIb	The abovementioned conditions in II are also observed in the domestic sphere.	Is unable to manage taking medication or stay alone at home due to an inability to answer the phone or the door.
III		Occasionally manifests communication difficulties or symptoms/behaviors that hinder daily activities, thus requiring care.	
	IIIa	Manifests abovementioned conditions described in III predominantly during the day.	Has difficulty or takes time to change clothes, take meals, defecate, or urinate; puts objects into the mouth, picks up and collects objects, is incontinent, makes loud and incoherent screams, carelessly handles fire, or engages in unhygienic acts or inappropriate sexual acts, etc.
	IIIb	Manifests abovementioned conditions described in III predominantly at night.	Same as rank IIIa.
IV		Frequently manifests difficulties communicating or symptoms/behaviors that hinder daily activities and constantly requires care.	Same as rank III.
M		Manifests significant mental symptoms, problematic behaviors, or severe physical illnesses and requires specialized medical care.	Shows continued mental symptoms, such as delirium, delusions, and agitation, and manifests associated problematic behaviors, such as self-mutilation or harm to others.

Table S2. Definitions of covariates

Variables	Original question and choice	Definition
**Demographic characteristics**		
Age	-	Continuous variable
Sex	-	Binary variable (0 = male, 1 = female)
Divorce or bereavement	-	Binary variable (0 = no, 1 = yes)
Living alone	-	Binary variable (0 = no, 1 = yes)
**Socioeconomic characteristics**		
Low educational attainment	-	Continuous variable (1 = ≧ 13 years, 2 = 10 to 12 years, 3 = 6 to 9 years, 4 = < 6 years)
Low equivalized household income	Equivalized household income was calculated as gross household income divided by the square root of household size and categorized into nine brackets using 0.5-million-yen intervals (≤1.5 million yen, 1.5–2.0 million yen, …, ≥5.0 million yen). In this study, the nine-category income scale was reverse-coded so that higher values indicated lower income.	Continuous variable
Unemployment	Q. What is your current employment status?<br>1. Currently employed<br>2. Retired and not currently employed<br>3. Never employed	Binary variable (0 = 1, 1 = 2/3)
No home ownership	Q. Which type of housing do you live in?<br>1. Owner-occupied housing<br>2. Privately rented housing<br>3. Public or semi-public rental housing (e.g., government or public corporation housing)<br>4. Company-provided housing or dormitory<br>5. Other	Binary variable (0 = 1, 1 = 2/3/4/5)
**Health conditions**		

Activity of daily living	Q. Can you perform activities such as walking, bathing, and toileting by yourself?<br>1. I can perform them independently without any assistance.<br>2. I can perform them only with some assistance, such as a helping hand.<br>3. I cannot perform them without complete assistance.	Ordinal scale
Depressive symptoms	Depressive symptoms were measured by GDS-15 (Geriatric Depression Scale-15). The score was categorized into lower (four points and under) versus higher (five points and over) risks.	Binary variable (0 = lower risk, 1 = higher risk)
Existing diseases or disabilities under medical treatments	Q. Do you have the following diseases or disabilities? (multiple answer)<br>1. Cancer<br>2. Heart disease<br>3. Stroke<br>4. High blood pressure<br>5. Diabetes<br>6. Hyperlipidemia<br>7. Arthritis and neuralgia<br>8. Injury / fracture<br>9. Respiratory disease<br>10. Impaired vision<br>11. Impaired hearing<br>12. Hepatic disease<br>13. Gastrointestinal disease<br>14. Obesity<br>15. Osteoporosis<br>16. Mental disorders<br>17. Urination disorders (e.g., incontinence, frequent urination, difficulty urinating, or urinary leakage)<br>18. Sleep disorders<br>19. Dysphagia (difficulty swallowing)	Binary variable (0 = No, 1 = Yes)
**Social relationship**		
Lack of receiving emotional social support	Q. Do you have someone who listens to your concerns and complaints?	Binary variable (0 = Yes, 1 = No)
Lack of providing emotional social support	Q. Do you listen to someone's concerns and complaints?	Binary variable (0 = Yes, 1 = No)

Lack of receiving instrumental social support	Q. Do you have someone who looks after you when you are sick and confined to a bed for a few days?	Binary variable (0 = Yes, 1 = No)
Lack of providing instrumental social support	Q. Do you look after someone when he/she is sick and confined to a bed for a few days?	Binary variable (0 = Yes, 1 = No)
Infrequency of participation in hobby clubs	Q. How often do you participate in volunteer clubs?<br>1. Almost everyday<br>2. Two or three times a week<br>3. Once a week<br>4. Once or twice a month<br>5. A few times a year<br>6. Never	Continuous variable
Infrequency of participation in sports clubs	Q. How often do you participate in sports clubs?<br>1. Almost everyday<br>2. Two or three times a week<br>3. Once a week<br>4. Once or twice a month<br>5. A few times a year<br>6. Never	Continuous variable
Infrequency of meeting friends	Q. How often do you see your friends?<br>1. Almost everyday<br>2. Two or three times a week<br>3. Once a week<br>4. Once or twice a year<br>5. A few times a year<br>6. Rarely	Continuous variable
Less daily social interaction with neighbors	Q. What kind of interactions do you have with people in your neighborhood?<br>1. Mutual consultation, lending and borrowing daily commodities, cooperation in daily life<br>2. Standing and chatting frequently<br>3. No more than exchanging greetings<br>4. None, not even greetings	Continuous variable
**Behavioral factor**		
Current smoking		Binary variable (0 = No, 1 = Yes)

Current drinking alcohol		Binary variable<br>(0 = No, 1 = Yes)
Infrequency of eating meat or fish in the past month	Q. How often did you eat meat or fish over the past month?<br>1. Twice a day or more<br>2. Once a day<br>3. Four to six times a week<br>4. Two or three times a week<br>5. Once a week<br>6. Less than once a week<br>7. None	Continuous variable
Infrequency of eating vegetables or fruits in the past month	Q. How often do you eat fruits and vegetables over the past month?<br>1. Twice a day or more<br>2. Once a day<br>3. Four to six times a week<br>4. Two or three times a week<br>5. Once a week<br>6. Less than once a week<br>7. None	Continuous variable
Infrequency of going out	Q. How often do you go out?<br>1. Almost everyday<br>2. Two or three times a week<br>3. Once a week<br>4. Once or twice a month<br>5. Several times a year<br>6. Rarely	Continuous variable
Shorter daily walking time	Q. How long do you walk a day on average?<br>1. Less than 30 minutes<br>2. 30 to 59 minutes<br>3. 60 to 89 minutes<br>4. 90 minutes or more	Continuous variable
Recent adverse life events (within the past year)		
Loss of a spouse		Binary variable<br>(0 = No, 1 = Yes)
Loss of relatives or friends		Binary variable<br>(0 = No, 1 = Yes)
Initiation of family caregiving		Binary variable<br>(0 = No, 1 = Yes)

**Environmental risk factor**		
	Q. How many of the following facilities are located within 1 kilometer of your home?<br>1. None<br>2. Few<br>3. Some<br>4. Many	
Places with graffiti or litter		Continuous variable
Parks and sidewalks suitable for exercise or walking		Continuous variable (reverse-coded; 1 = Many ~ 4 = None)
Places that are difficult to walk due to hills or steps		Continuous variable
Roads or intersections with a high risk of traffic accidents		Continuous variable
Attractive scenery or buildings		Continuous variable (reverse-coded; 1 = Many ~ 4 = None)
Places where walking alone at night feels unsafe		Continuous variable

Table S3. Descriptive statistics of baseline covariates (not included in Table 1)

	n (%)/mean (SD)
**Health conditions**	
Activity of daily living (1: Independent to 3: Completely dependent), mean (SD)	1.02 (0.16)
Missing, n (%)	975 (2.9)
Depressive symptoms (>=5 points of GDS-15 score), n (%)	7,623 (22.5)
Missing, n (%)	6,015 (17.8)
Existing diseases or disabilities under medical treatments	
Cancer, n (%)	1,512 (4.5)
Missing, n (%)	8,143 (24.0)
Heart disease, n (%)	4,083 (12.1)
Missing, n (%)	8,143 (24.0)
Stroke, n (%)	419 (1.2)
Missing, n (%)	8,143 (24.0)
High blood pressure, n (%)	13,124 (38.7)
Missing, n (%)	8,143 (24.0)
Diabetes, n (%)	4,181 (12.3)
Missing, n (%)	8,143 (24.0)
Hyperlipidaemia, n(%)	3,361 (9.9)
Missing, n (%)	8,143 (24.0)
Arthritis and neuralgia, n (%)	3,659 (10.8)
Missing, n (%)	8,143 (24.0)
Injury / fracture, n (%)	456 (1.3)
Missing, n (%)	8,143 (24.0)
Respiratory disease, n (%)	1,273 (3.8)
Missing, n (%)	8,143 (24.0)
Impaired vision, n (%)	4,474 (13.2)
Missing, n (%)	8,143 (24.0)
Impaired hearing, n (%)	2,300 (6.8)
Missing, n (%)	8,143 (24.0)
Hepatic disease, n (%)	653 (1.9)
Missing, n (%)	8,143 (24.0)
Gastrointestinal disease, n (%)	2,029 (6.0)
Missing, n (%)	8,143 (24.0)

Obesity, n (%)	1,211 (3.6)
Missing, n (%)	8,143 (24.0)
Osteoporosis, n (%)	2,352 (6.9)
Missing, n (%)	8,143 (24.0)
Mental disorders, n (%)	321 (0.9)
Missing, n (%)	8,143 (24.0)
Urination disorders (e.g., incontinence, frequent urination, difficulty urinating, or urinary leakage), n (%)	2,446 (7.2)
Missing, n (%)	8,143 (24.0)
Sleep disorders, n (%)	2,143 (6.3)
Missing, n (%)	8,143 (24.0)
Dysphagia (difficulty swallowing), n (%)	197 (0.6)
Missing, n (%)	8,143 (24.0)
**Social relationship**	
Lack of receiving emotional social support, n (%)	1,954 (5.8)
Missing, n (%)	1,955 (5.8)
Lack of providing emotional social support, n (%)	2,256 (5.7)
Missing, n (%)	2,302 (6.8)
Lack of receiving instrumental social support, n (%)	1,814 (5.4)
Missing, n (%)	1,873(5.5)
Lack of providing instrumental social support, n (%)	3,838 (11.3)
Missing, n (%)	2,811 (8.3)
Infrequency of participation in hobby clubs (1: Almost everyday to 6: Never), mean (SD)	4.74 (1.54)
Missing, n (%)	6,449 (19.0)
Infrequency of participation in sports clubs (1: Almost everyday to 6: Never), mean (SD)	5.13 (1.52)
Missing, n (%)	7,604 (22.4)
Infrequency of meeting friends (1: Almost everyday to 6: Rarely), mean (SD)	3.26 (1.55)
Missing, n (%)	2,499 (7.4)
Less daily social interaction with neighbors (1: Mutual consultation to 4: None), mean (SD)	2.16 (0.68)
Missing, n (%)	2,914 (8.6)
**Behavioral factors**	
Current smoking n (%)	3,462 (10.2)
Missing, n (%)	3,362 (9.9)
Current drinking alcohol n (%)	11,516 (34.0)

Missing, n (%)	2,213 (6.5)
Infrequency of eating meat or fish in the past month (1: Twice a day or more to 7: None), mean (SD)	2.94 (1.17)
Missing, n (%)	2,222 (6.6)
Infrequency of eating vegetables or fruits in the past month (1: Twice a day or more to 7: None), mean (SD)	1.84 (1.01)
Missing, n (%)	2,005 (5.9)
Infrequency of going out (1: Almost everyday to 6: Rarely), mean (SD)	1.66 (0.98)
Missing, n (%)	2,020 (6.0)
Shorter walking time per day (1: Less than 30 minutes to 4: 90 minutes or more), mean (SD)	2.85 (1.05)
Missing, n (%)	2,178 (6.5)
**Recent adverse life events (within the past year)**	
Loss of a spouse, n (%)	758 (2.2)
Missing, n (%)	2,384 (7.0)
Loss of relatives or friends, n (%)	8,961 (26.5)
Missing, n (%)	2,384 (7.0)
Initiation of family caregiving, n (%)	1,236 (3.6)
Missing, n (%)	2,384 (7.0)
**Environmental risk factor**	
Places with graffiti or litter (reverse-coded; 1 = None ~ 4 = Many), n (%)	2.22 (0.73)
Missing, n (%)	3,796 (11.2)
Parks and sidewalks suitable for exercise or walking, n (%)	2.13 (0.73)
Missing, n (%)	2,091 (6.2)
Places that are difficult to walk due to hills or steps (reverse-coded; 1 = None ~ 4 = Many), n (%)	2.40 (0.76)
Missing, n (%)	1,934 (5.7)
Roads or intersections with a high risk of traffic accidents (reverse-coded; 1 = None ~ 4 = Many), n (%)	2.82 (0.67)
Missing, n (%)	1,995 (5.9)
Attractive scenery or buildings, n (%)	2.66 (0.74)
Missing, n (%)	3,144 (9.3)
Places where walking alone at night feels unsafe (reverse-coded; 1 = None ~ 4 = Many), n (%)	2.79 (0.66)
Missing, n (%)	3,590 (10.6)

Abbreviation: GDS-15, Geriatric Depression Scale-15

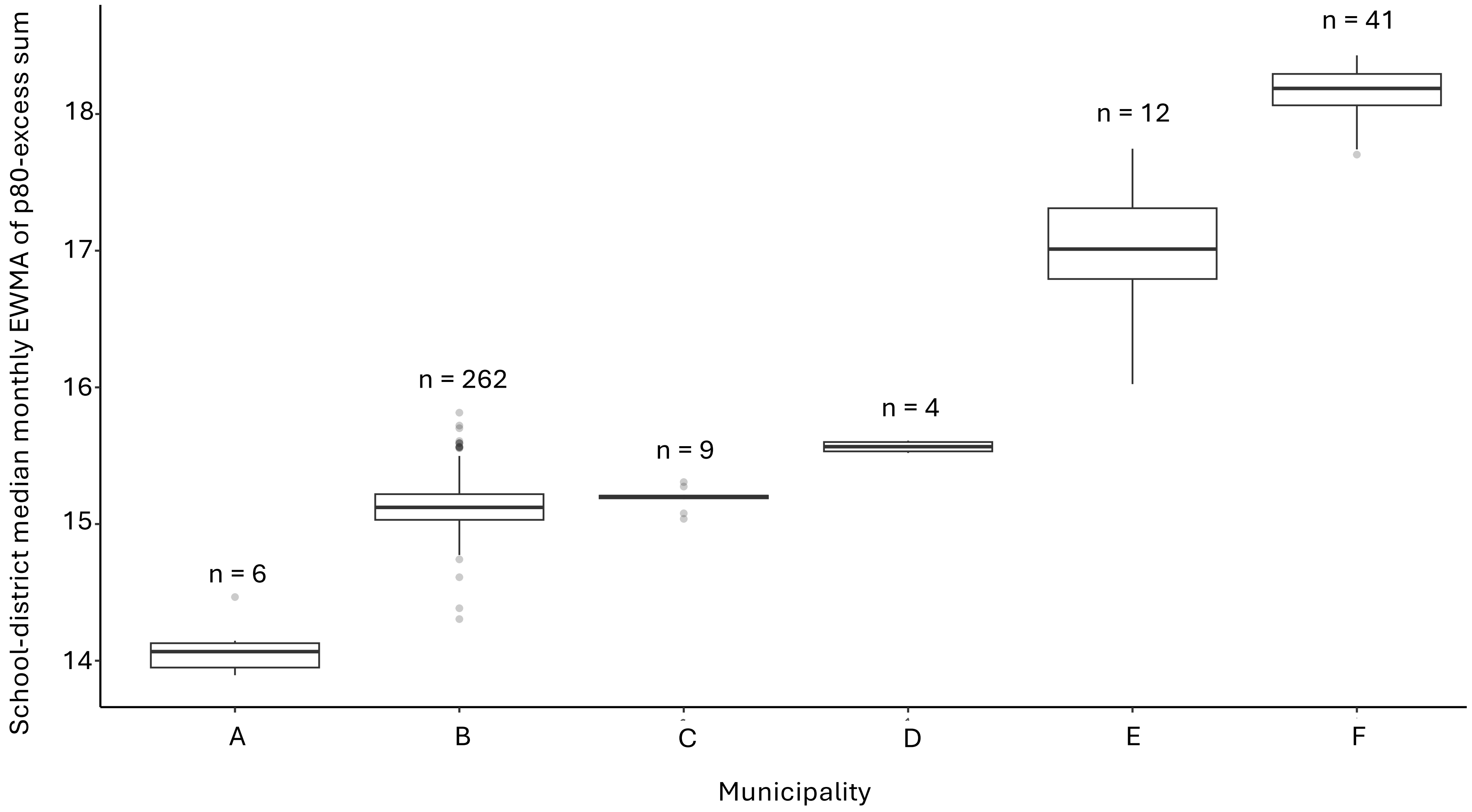

Figure S1. Spatial variability in school-district heat exposure within municipalities (EWMA 24-month; half-life = 12 months)

Note: Labels above the boxes indicate the number of school districts per municipality.

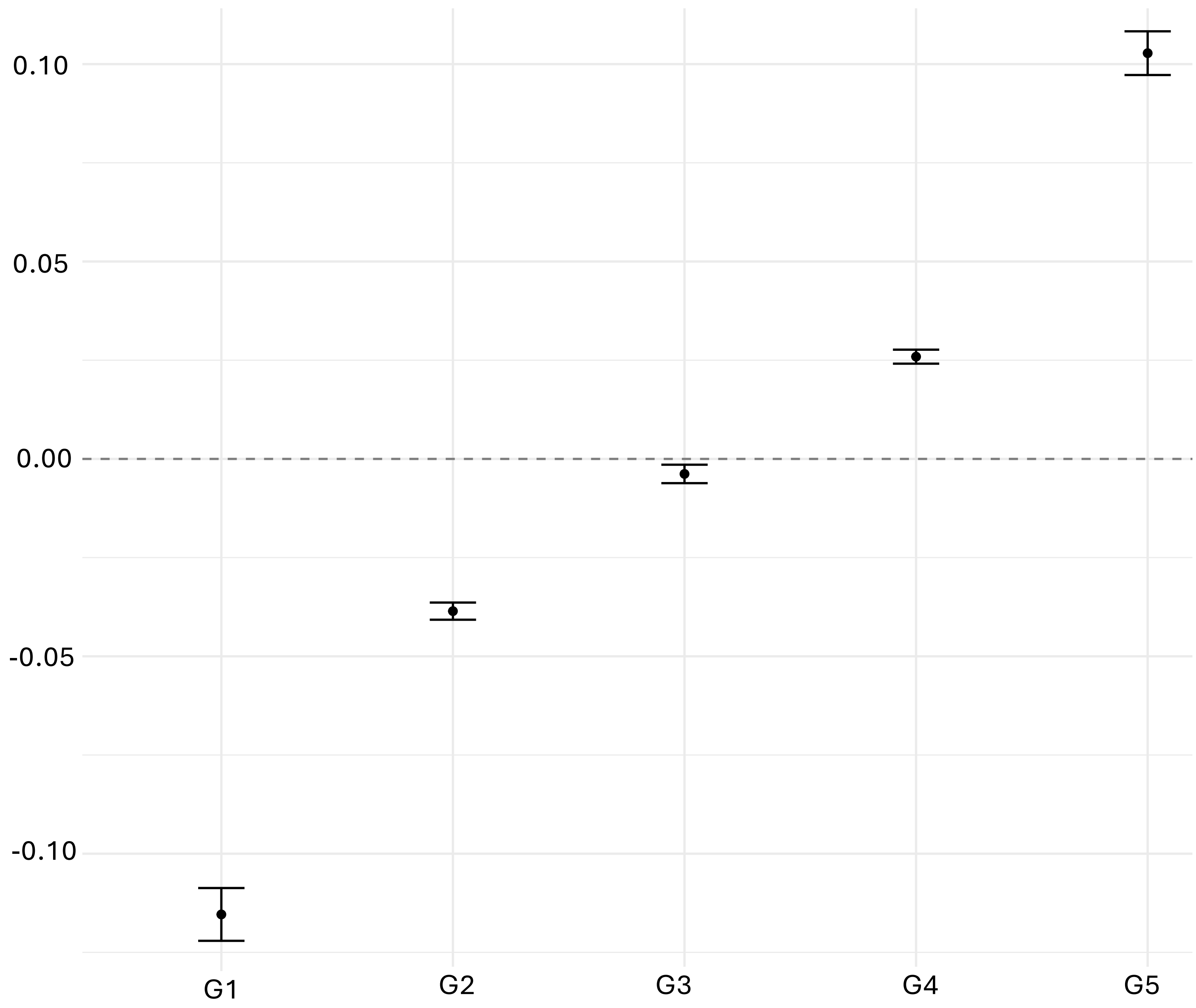

Figure S2. Group average treatment effects (GATES) across five CATE quantile groups

Table S4. R-learner component comparison across CATE strata

	Negative CATE (<−0.002)	Near-zero CATE (−0.002 to 0.002)	Positive CATE (>0.002)
n [a]	12,782	6,229	12,995
$\hat{m}$ median [b]	0.002	0.001	0.002
Y median [c]	-0.001	-0.001	-0.001
$\tilde{T}$ median [d]	+0.018	≈0	-0.018

[a] Of the 33,877 participants at baseline, 1,871 were excluded because their follow-up period was insufficient to complete the 24-month EWMA exposure window, leaving 32,006 participants for the CATE analysis.

[b] Predicted outcome (cognitive decline risk)

[c] Outcome residual (observed minus predicted outcome)

[d] Exposure residual (observed minus predicted cumulative heat exposure).

Note: Negative values of $\tilde{T}$ indicate that individuals received less heat exposure than their covariate profile would predict; positive values indicate the reverse. CATE, conditional average treatment effect; EWMA, exponentially weighted moving average.

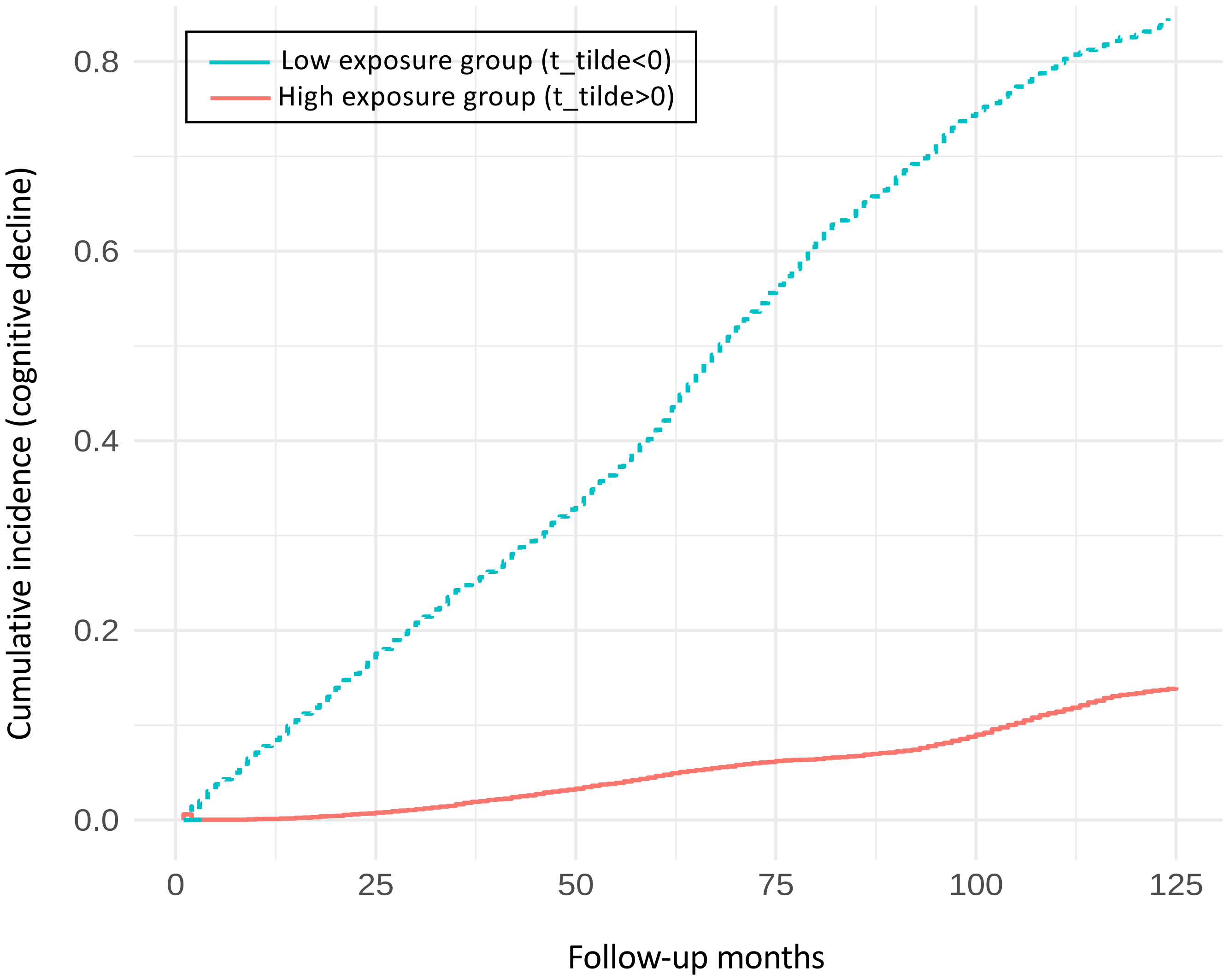

Figure S3. Cumulative incidence of cognitive decline by exposure residual subgroup within the negative CATE group

Note: Cumulative incidence functions for cognitive decline are shown for two subgroups within the negative conditional average treatment effect (CATE) group, defined by the sign of the exposure residual ($\tilde{T}$). The high exposure group ($\tilde{T} > 0$) comprises individuals who received more cumulative heat exposure than their covariate profile would predict. The low exposure group ($\tilde{T} \leq 0$) comprises individuals who received less cumulative heat exposure than predicted. Competing risks (death and residential transfer) were accounted for in the estimation. CATE, conditional average treatment effect.

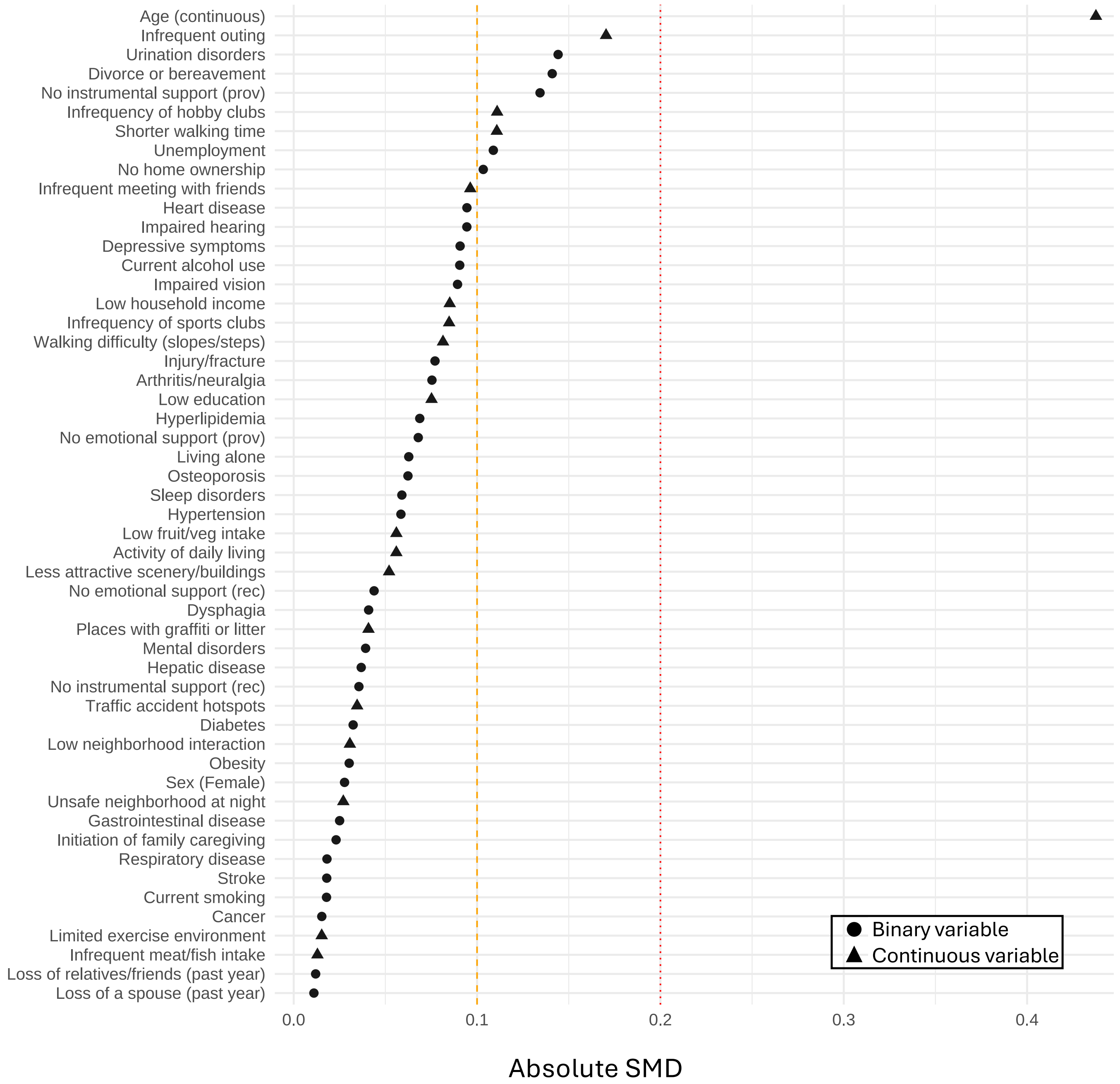

Figure S4. Baseline covariate balance between exposure residual subgroups within the negative CATE group

Note: Standardized mean differences (SMDs) in baseline characteristics between the high exposure group ($\tilde{T} > 0$) and the low exposure group ($\tilde{T} \leq 0$) within the negative CATE group. An SMD below 0.10 indicates good balance between groups.

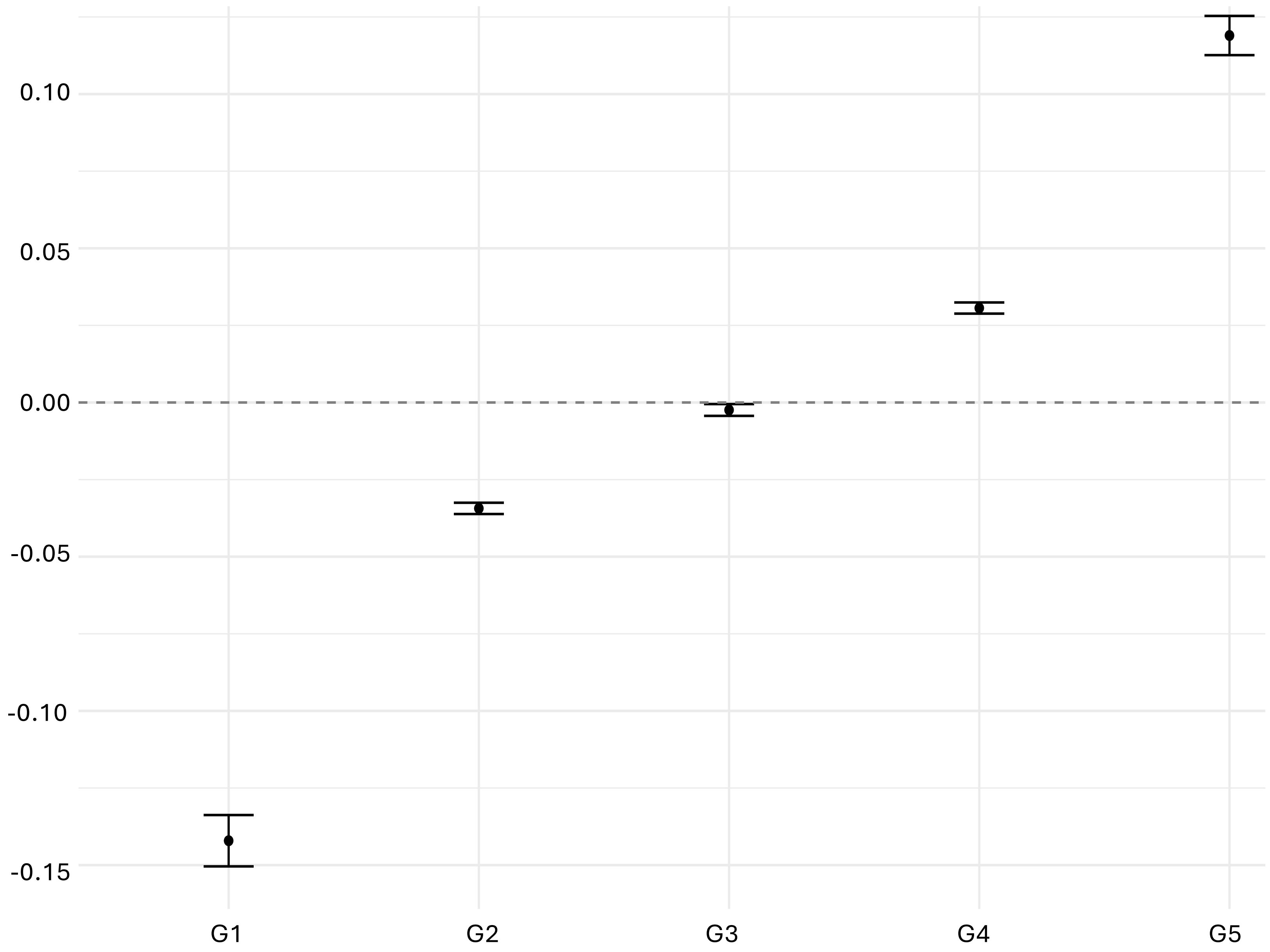

Figure S5. Group average treatment effects (GATES) across five CATE quantile groups: sensitivity analysis with a 36-month exposure lag

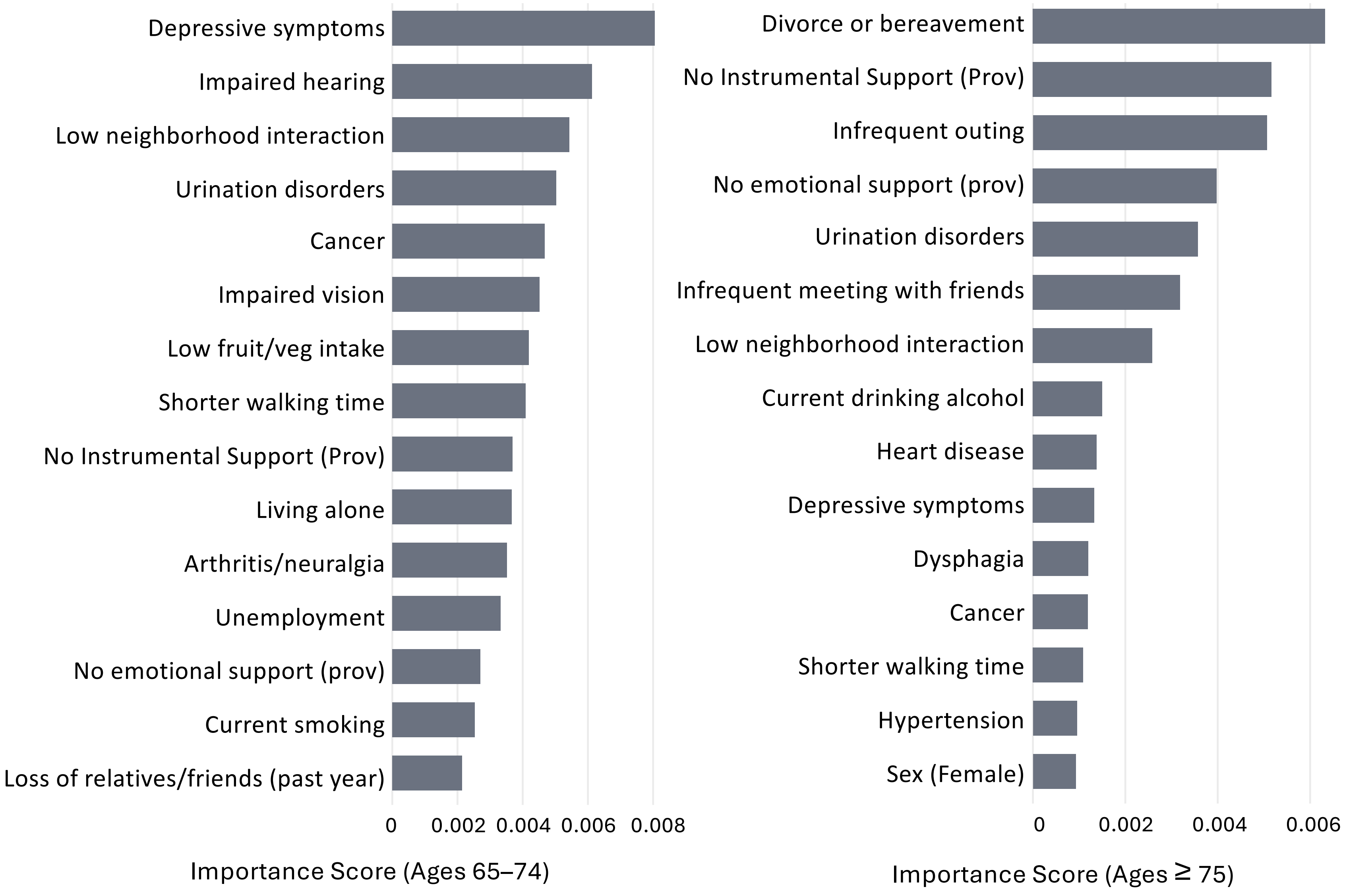

Figure S6. Permutation importance of vulnerability factors for high heat susceptibility (CATE ≥ 80th percentile), by age stratum: sensitivity analysis with a 36-month exposure lag

Note: Importance scores represent the decrease in area under the receiver operating characteristic curve (ΔAUC) when each variable is randomly permuted. Higher values indicate greater discriminative contribution to identifying individuals above the 80th percentile of the positive-CATE distribution. CATE, conditional average treatment effect; ΔAUC, change in area under the receiver operating characteristic curve.

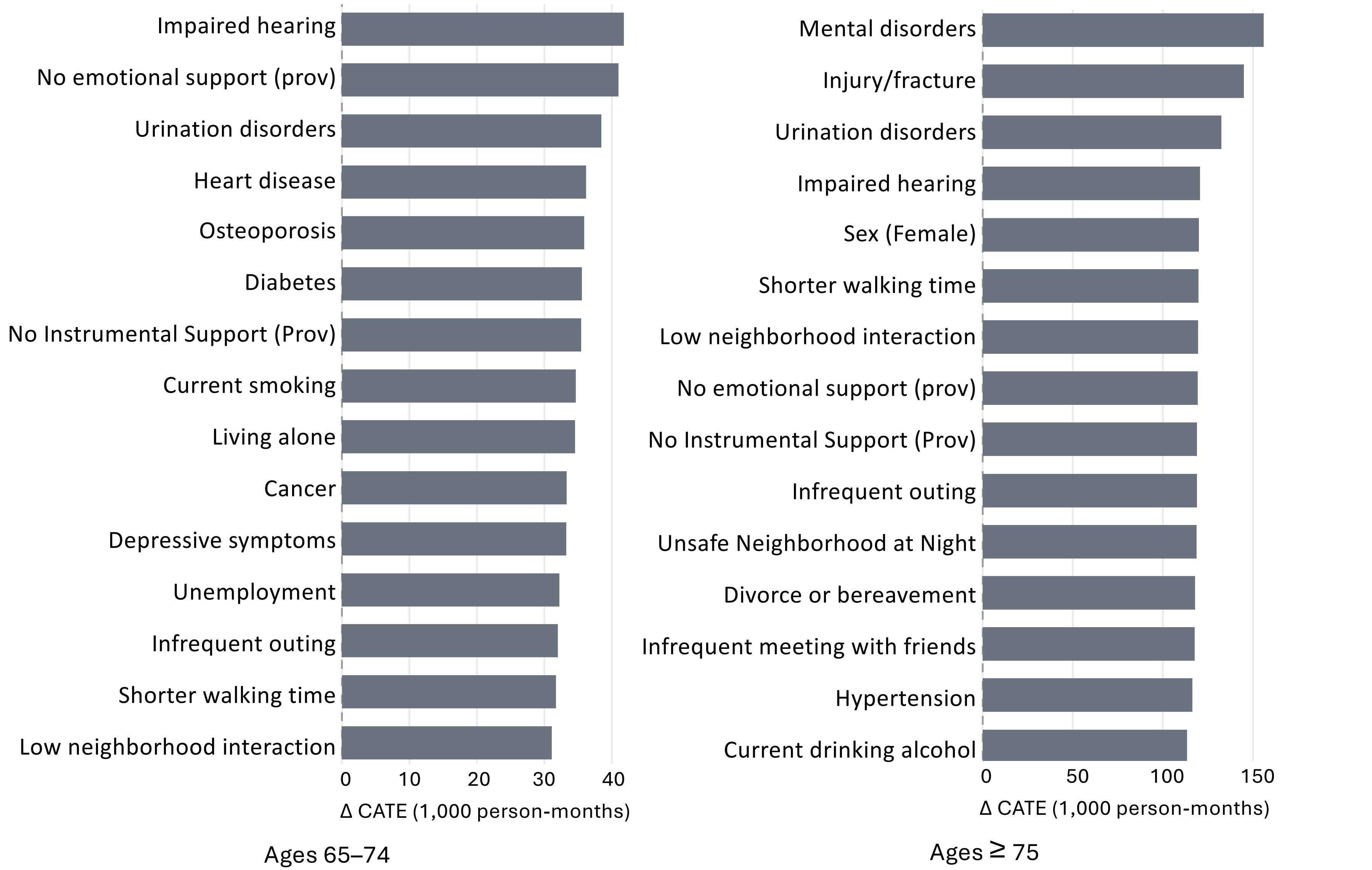

Figure S7. Excess CATE above the 80th percentile threshold for the top 15 vulnerability attributes, by age stratum: sensitivity analysis with a 36-month exposure lag

Note: The mean excess CATEs (Δ) estimated within the high-vulnerability subpopulation (CATE ≥ 80th percentile). CATE, conditional average treatment effect.

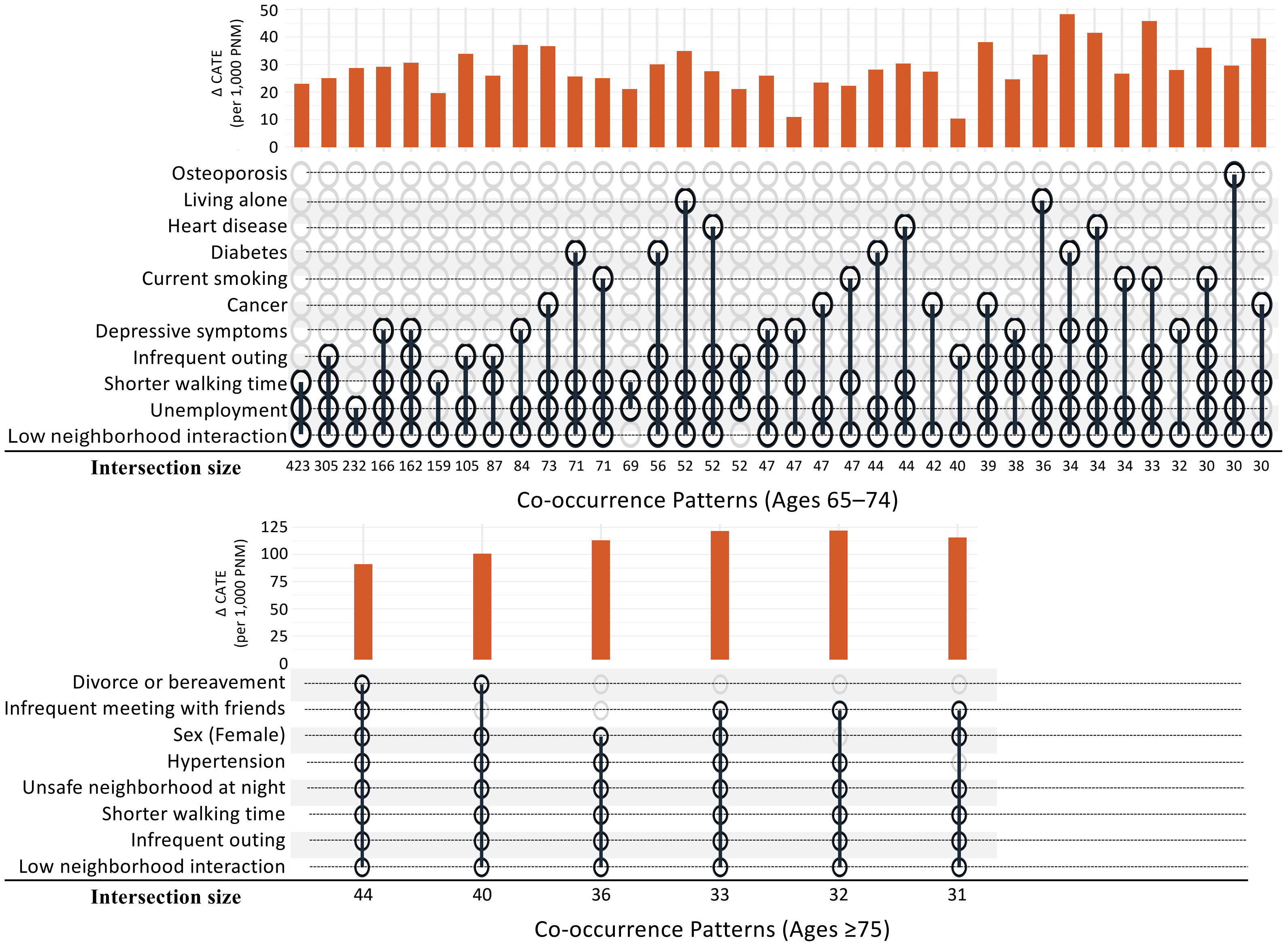

Figure S8. Co-occurrence patterns of vulnerability attributes and mean excess CATE within the high-vulnerability subpopulation (CATE ≥ 80th percentile), by age stratum: sensitivity analysis with a 36-month exposure lag

Note: CATE, conditional average treatment effect; PNM, person-months.

Supplementary Methods

・Full details of Distributed Lag Non-Linear Models

We identified the most appropriate lag structure and heat exposure specification using DLNMs within Poisson generalized additive models (GAMs) [1], fitted to person-month data separately for each municipality using the dlnm and mgcv packages. The estimated exposure–lag associations were then pooled using multivariate random-effects meta-analysis implemented in the mvmeta package. This two-stage approach accommodates municipality-specific exposure–response functions while accounting for between-municipality heterogeneity [2]. We compared candidate models across three dimensions: WBGT exceedance thresholds (80th, 85th, and 90th percentiles), maximum lag periods (12, 18, 24, 30, and 36 months), and spline complexity (2 to 4 degrees of freedom for both exposure and lag). Each municipality-specific model was adjusted for long-term trends using a thin-plate regression spline for calendar month (basis dimension k = 10) over the 13-year study period [3], as well as age, sex, educational attainment, equivalized household income, employment status, marital status, depressive symptoms, and comorbidities (heart disease and stroke). The exposure–response association was centered at zero exceedance, representing no exceedance above the school district–specific WBGT threshold, which served as the reference level. Model selection was based primarily on the quasi-Akaike information criterion, with consideration of epidemiological interpretability [1]. The final specification comprised the monthly sum of population-weighted WBGT exceedances above the 80th percentile, a 24-month lag, and natural splines with 2 degrees of freedom for exposure and 4 degrees of freedom for lag.

Reference

[1] Gasparrini A, Armstrong B, Kenward MG. Distributed lag non-linear models. Stat Med 2010;29:2224–34. https://doi.org/10.1002/sim.3940.

[2] Gasparrini A, Armstrong B. Reducing and meta-analysing estimates from distributed lag non-linear models. BMC Med Res Methodol 2013;13:1. https://doi.org/10.1186/1471-2288-13-1.

[3] Wood SN. Thin Plate Regression Splines. J R Stat Soc Ser B Stat Methodol 2003;65:95–114. https://doi.org/10.1111/1467-9868.00374.
